# Supplementary material for: Histone H4K20 methylation mediated chromatin compaction threshold ensures genome integrity by limiting DNA replication licensing
Source: Nat Commun. 2018 Sep 12;9:3704. doi: 10.1038/s41467-018-06066-8 (PMC6135857; doi:10.1038/s41467-018-06066-8)
Supplement: Supplementary file 1 — Supplementary Information [file 41467_2018_6066_MOESM1_ESM.pdf]

Supplementary information

Histone H4K20 methylation mediated chromatin compaction threshold ensures genome integrity by limiting DNA replication licensing

Shoaib, *et al*

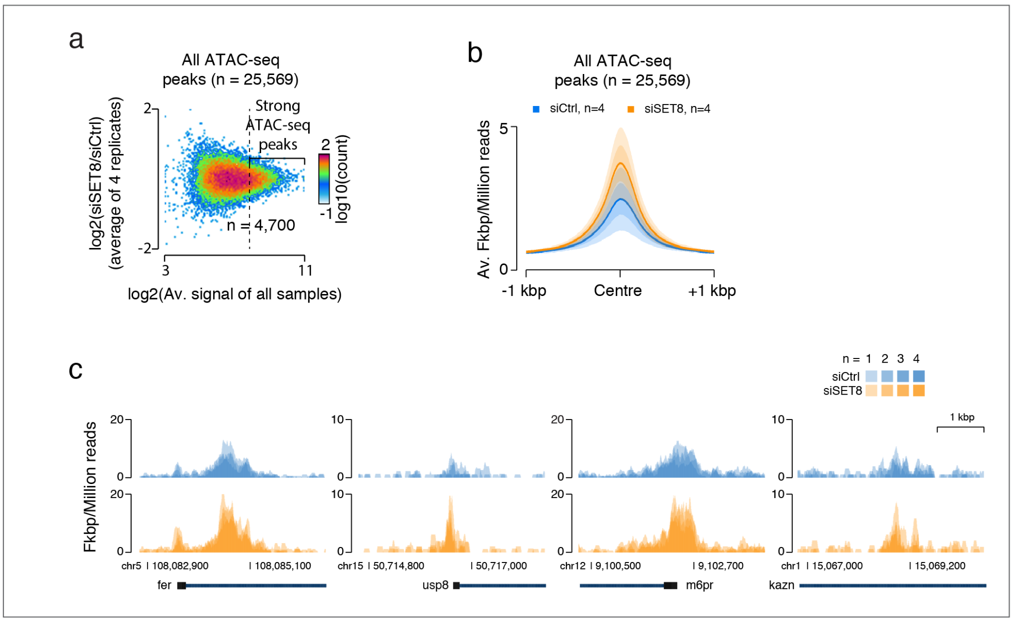

Supplementary Figure 1. ATAC-seq shows genome-wide chromatin compaction changes in SET8-kd cells. **(a)** U2OS cells were synchronized with double thymidine block and siRNA transfected with siControl or siSET8 6h (hours) before G1/S release. Cells were harvested and subjected to the ATAC-seq protocol as described in materials and methods. 2D-histogram of average ATAC-seq signals in control and siSET8 (x-axis, n = 8) and log2 fold difference between the averages of quantile normalized siSET8 (n = 4) and averages of quantile normalized siControl (n = 4) ATAC-seq signal in U2OS cells at 25,569 sites with elevated chromatin openness. Color coding reflects the log10 count of loci with a given combination of values. The vertical dashed line depicts the threshold used for defining the subpopulation of loci with strong ATAC-seq signal. X-axis values are quantile normalized read counts within 1kbp of each locus. **(b)** Plot of average ATAC-seq signal at the surrounding 1 kbp of 25,569 loci in siSET8 and siControl cells. Lightly stained areas illustrate values for 1 and 2 standard deviations from the mean. Y-axis values are average Fragments per kbp per Million reads. **(c)** Transparent tracks of four loci illustrating typical ATAC-seq signal distributions at and around loci with strong ATAC-seq signal in siSET8
(orange) and siControl (blue) U2OS cells. Hg19 coordinates and genes annotated in Refseq are shown below. Y-axis values are Fragments per kbp per Million reads.

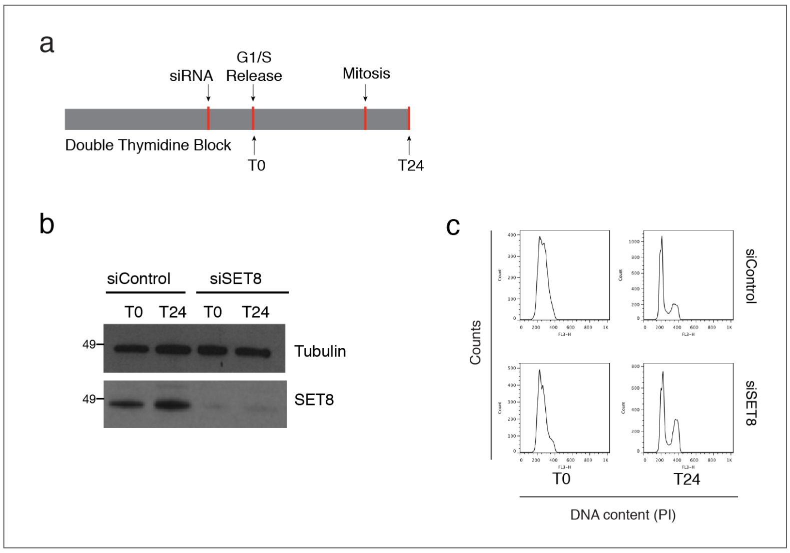

Supplementary Figure 2. Schematics, FACS profiles and immunoblot validations for FLIM-FRET experiment described in Fig. 1e. **(a)** Design of the experiment. H2B-2FP expressing U2OS cells were synchronized with single thymidine block and transfected with siControl and siSET8 at G1/S border. FRET was subsequently measured either immediately after release (T0) or in next G1 phase (T24). **(b)** Immunoblots of the samples in (Fig. 1e) with indicated antibodies. **(c)** Flow cytometry profiles of the samples in (Fig. 1e) stained with propidium iodide (PI) for DNA.
n>10,000.

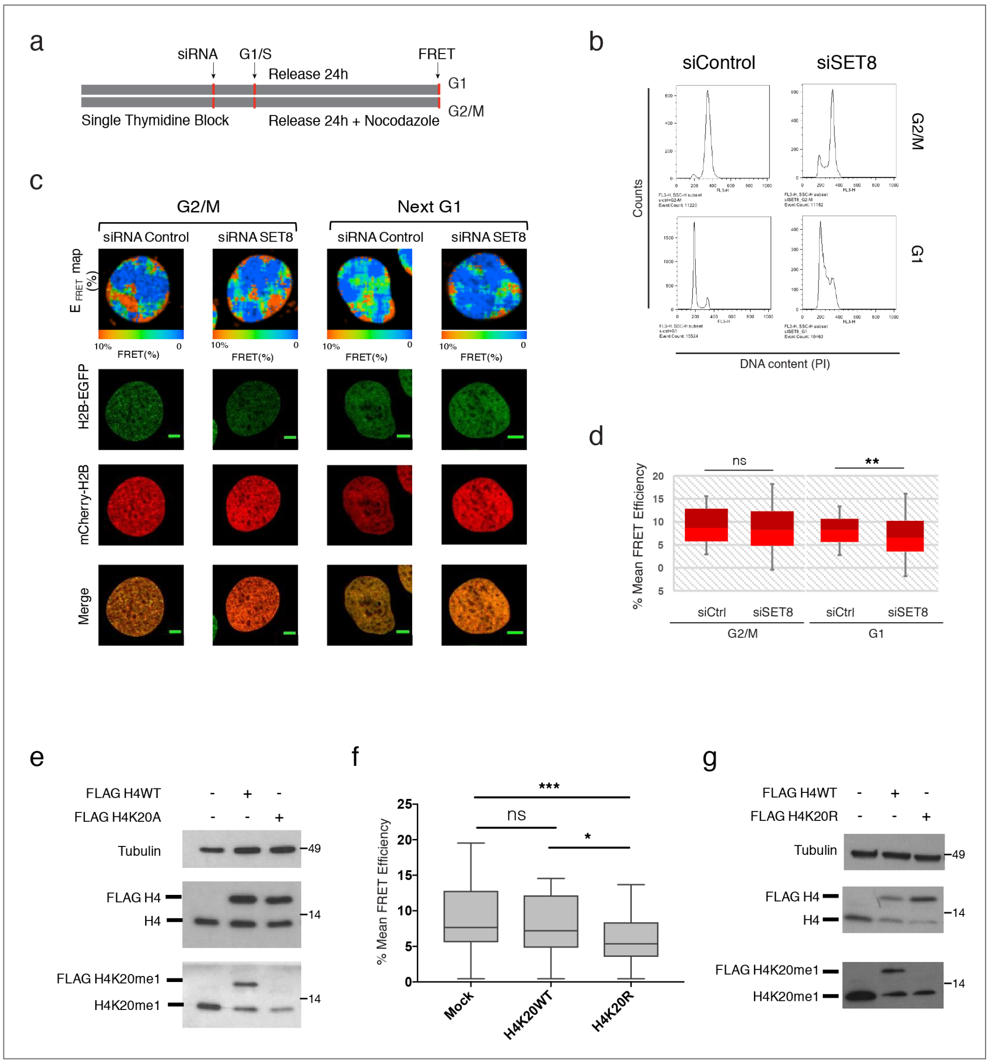

Supplementary Figure 3. FLIM-FRET analysis of U2OS$_{H2B-2FPs}$ shows chromatin compaction changes in siSET8 treated cells as well as histone H4 lysine 20 to alanine or arginine mutant expressing cells. **(a)** Experimental design. U2OS cells stably expressing H2B-GFP alone (U2OS$_{H2B-GFP}$) or with mCherry tagged histone H2B (U2OS$_{H2B-2FPs}$) were synchronized with single thymidine block and transfected with siControl and siSET8 at the G1/S border. Cells were then released in the presence or absence of nocodazole and FRET was measured 24h later. Cells with nocodazole were arrested in mitosis while cells release without nocodazole progressed into the next G1 phase. **(b)** Flow cytometry profile of the samples in (Supplementary fig. 2a) stained with PI for DNA content. n>10,000. **(c)** FRET measurements of samples from (Supplementary fig. 2a) were taken 24h after release (Bar, 10 µm). **(d)** Quantification of the FLIM–FRET chromatin compaction assay. Inside box-plots, the thick line represents median, the limit of the boxes corresponds to the mean FRET values that are upper or lower to the median, with whiskers extending to the highest and lowest FRET value. n>30 nuclei. **p<0.01 (ANOVA), ns (not significant). **(e)** Immunoblots of the samples in (Fig. 1i) probed with the indicated antibodies. **(f)** U2OS cells stably expressing H2B-GFP alone (U2OS$_{H2B-GFP}$) or with mCherry tagged histone H2B (U2OS$_{H2B-2FPs}$) were transduced with FLAG-tagged Histone H4WT or H4K20R mutant. Mock transduced cells were taken as control. Quantification of the mean FRET levels in Mock, H4K20WT and H4K20A expressing cells. Inside box-plots, the thick line represents median, the limit of the boxes corresponds to the mean FRET values that are upper or lower to the median, with whiskers extending to the highest and lowest FRET value. n>30 nuclei. ***p<0.001 (ANOVA), *p<0.05 (ANOVA), ns (not significant). **(g)** Immunoblots of the samples in (Supplementary fig. 2f) probed with the indicated antibodies.

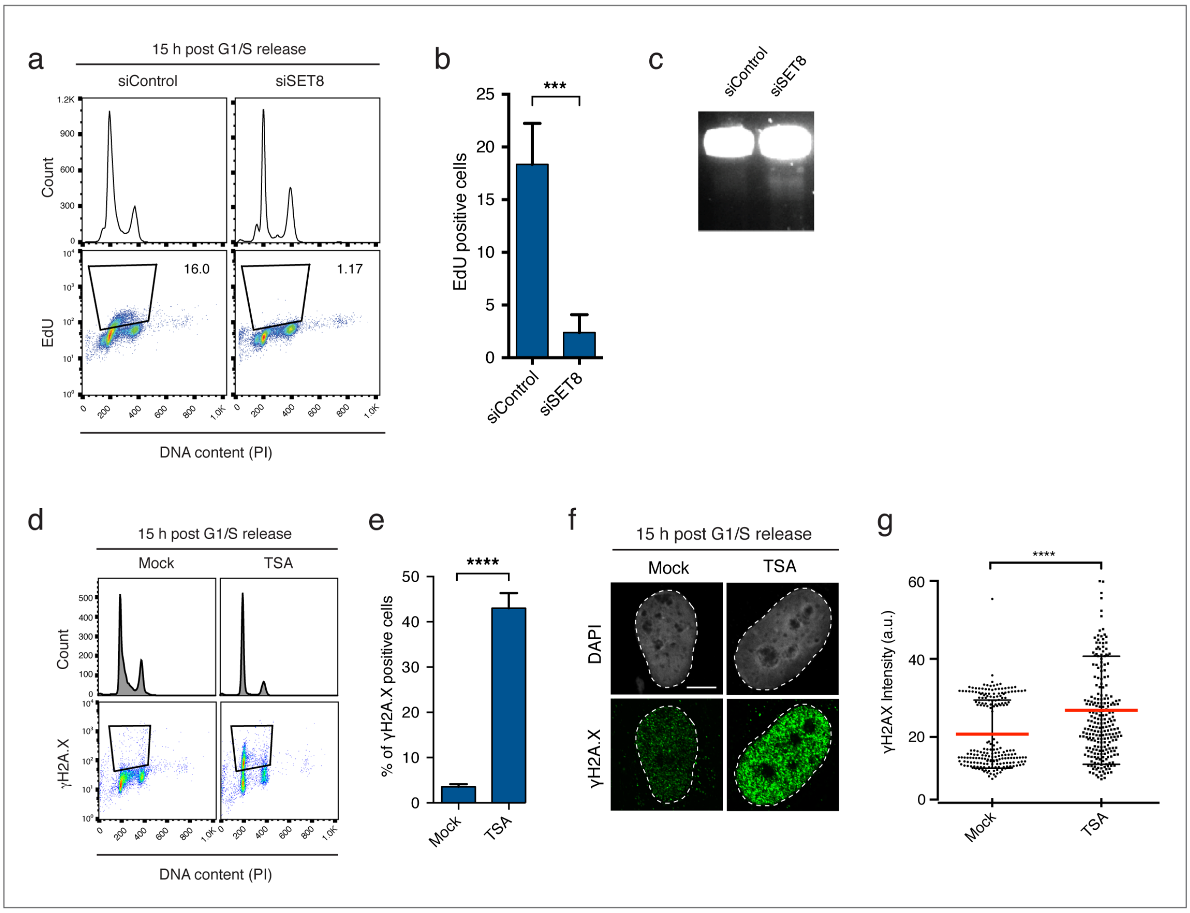

Supplementary Figure 4. TSA (HDAC inhibitor) treated U2OS cells mimics DNA damage observed in siSET8 treated cells. **(a)** U2OS cells were synchronized by double thymidine block and were depleted of SET8 during the second block, 6h before release from G1/S boundary. Cells were then pulse labelled with EdU for 30 min at 15h from G1/S release. Cells were then collected and subjected to Click-it reaction and PI staining before analysis by flow cytometry. **(b)** Bars represent percentage of EdU positive cells in the indicated samples (from Supplementary figure 4a, average ± SD of 3 independent experiments). ***p<0.001 (unpaired t-test). n>10,000 in each experiment. **(c)** U2OS cells were synchronized by double thymidine block and were transfected with Control and SET8 siRNA 6h before release from the G1/S boundary. Cells were then harvested at 15h post G1/S release and processed for PFGE analysis. **(d)** U2OS cells were synchronized by double thymidine block and treated with TSA at 12 h post G1/S release for a total of 3h. Graph shows flow cytometry profiles of the samples in stained with γH2A.X antibody and PI for DNA content. **(e)** Bars represent percentage of γH2A.X positive cells in
(Supplementary fig. 4d). Average ± SD of 3 independent experiments. ****p<0.0001 (unpaired t-test). n>20,000 in each experiment. **(f)** U2OS cells were synchronized and treated as in (Supplementary fig. 4d). 15h post G1/S release cells were fixed and stained with DAPI and γH2A.X antibody. (Bar, 10 μm). **(g)** Bars represent percentage of γH2A.X positive cells in (Supplementary fig. 4f). Average ± SD indicated by solid lines. ****p<0.0001 (unpaired t-test). n>150.

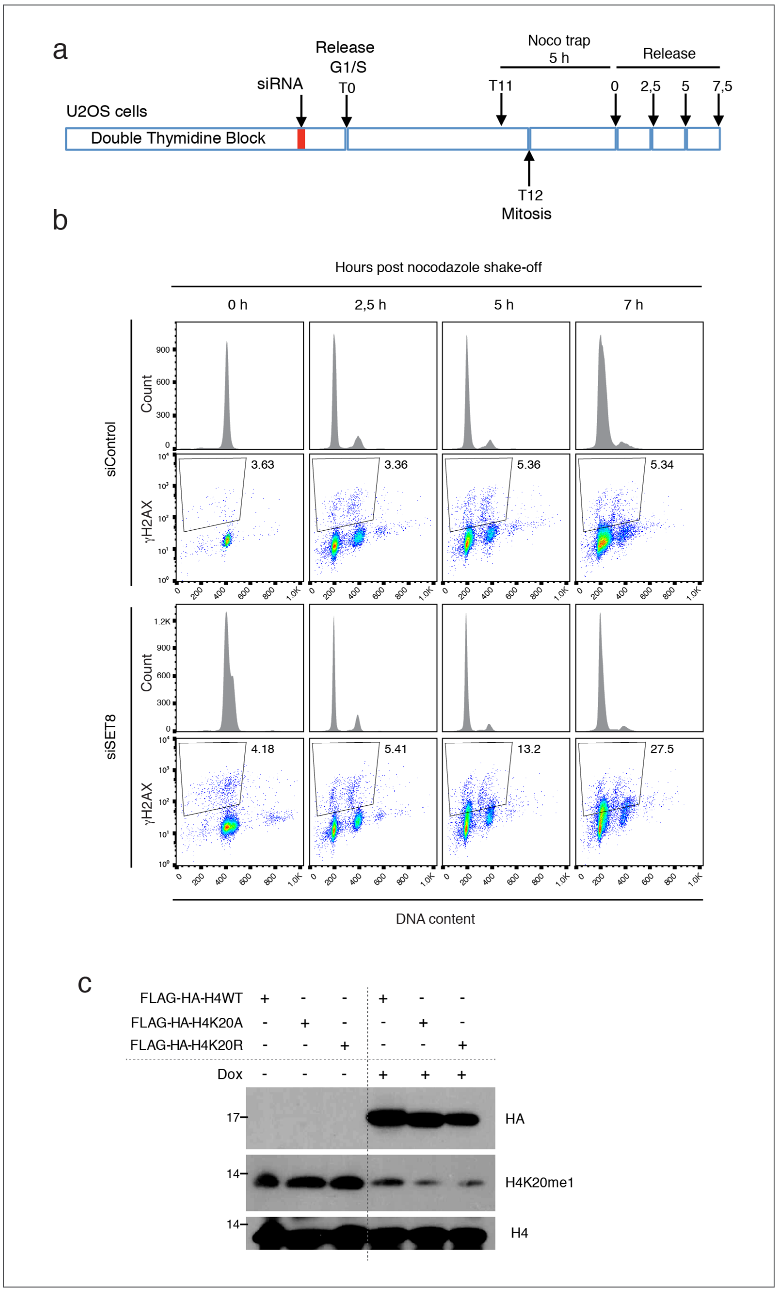

Supplementary Figure 5. DNA damage arise in siSET8 treated cells as the cells approach S phase. **(a)** Design of the experiment. U2OS cells were synchronized by double thymidine block and were depleted of SET8 during the second block, 6h before release from the G1/S boundary. Cells were treated with Nocodazole 11h after thymidine release. 5h later, cells were released from the Nocodazole trap and were collected at indicated time points. **(b)** Cells from (a) were fixed for flow cytometry analysis. Samples were stained with γH2A.X antibody and PI for DNA content. n>15,000. **(c)** Chromatin fractions from H4K20WT/A/R expressing cell lines with and without doxycycline, immunoblotted with the indicated antibodies.

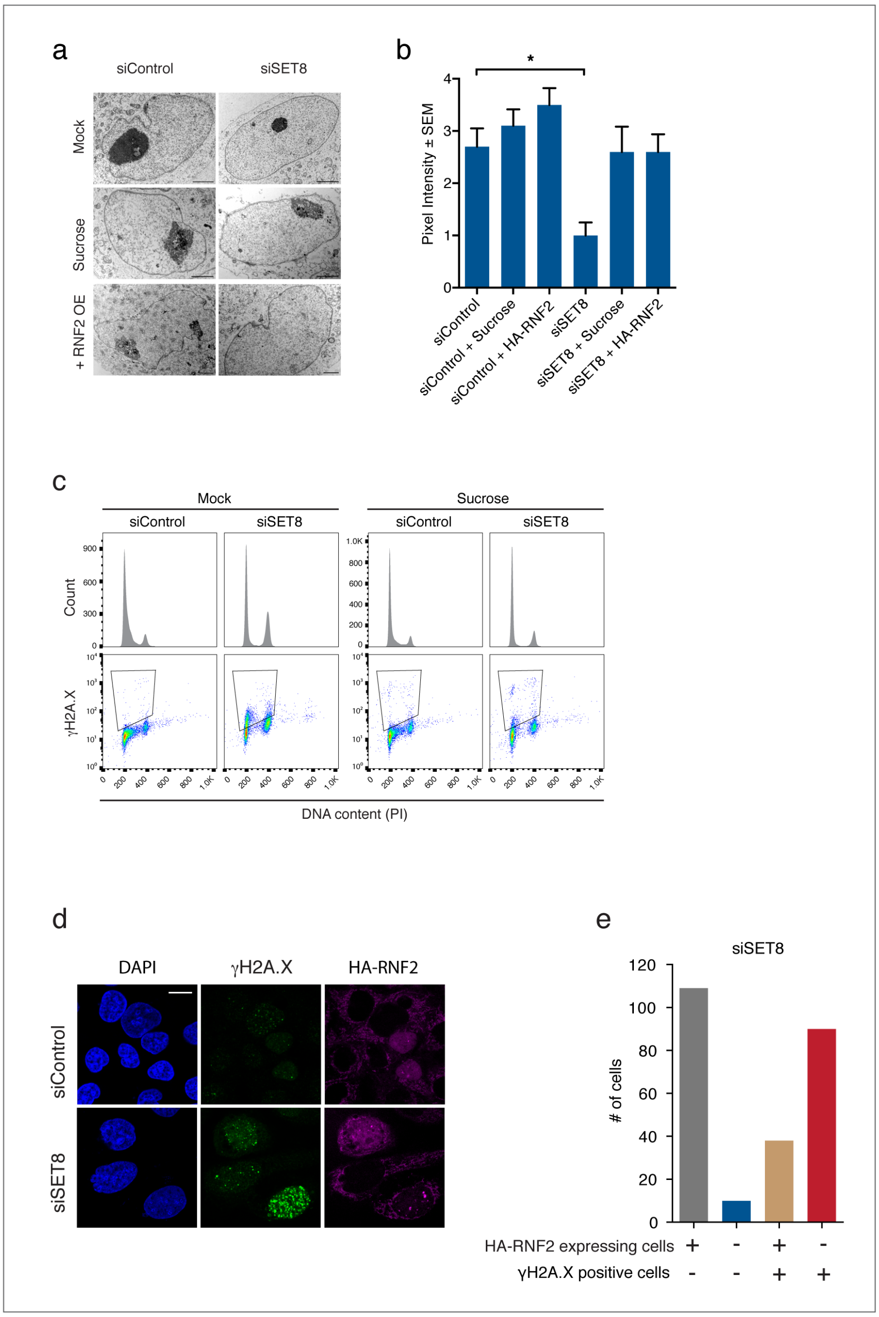

Supplementary Figure 6. Ectopic expression of RNF2 and addition of sucrose based hypertonic medium compacts chromatin and suppresses genome instability. **(a)** U2OS cells synchronized with a double thymidine block and siRNA transfected 6h before G1/S release. Cells were fixed at 15h post release for scanning electron microscope visualization as described in materials and methods. FLAG-HA-RNF2 was transiently over expressed at the start of the experiment. Sucrose was added at 12 h post G1/S release for a total of 3h until fixation at 15h. **(b)** Bars represent quantification of nuclei fixed for electron microscope visualization in (a). Mean pixel intensity ± SEM, n>15 nuclei). *p<0.05 (ANOVA). **(c)** Graph shows flow cytometry profiles of the samples in Fig 2j, stained with γH2A.X antibody and PI for DNA content. n>15,000. **(d)** U2OS cells were transfected with FLAG-HA-tagged human wild-type RNF2 (RING1b) and were double thymidine synchronized and treated with either siControl or siSET8 siRNAs as previously. Cells were fixed at 15h post G1/S release and stained with the indicated antibodies. (Bar, 20 μm). **(e)** Histogram represents the number of HA and γH2A.X positive cells in siSET8 treated sample. Majority of cells expressing FLAG-HA-tagged RNF2 were negative for γH2A.X.

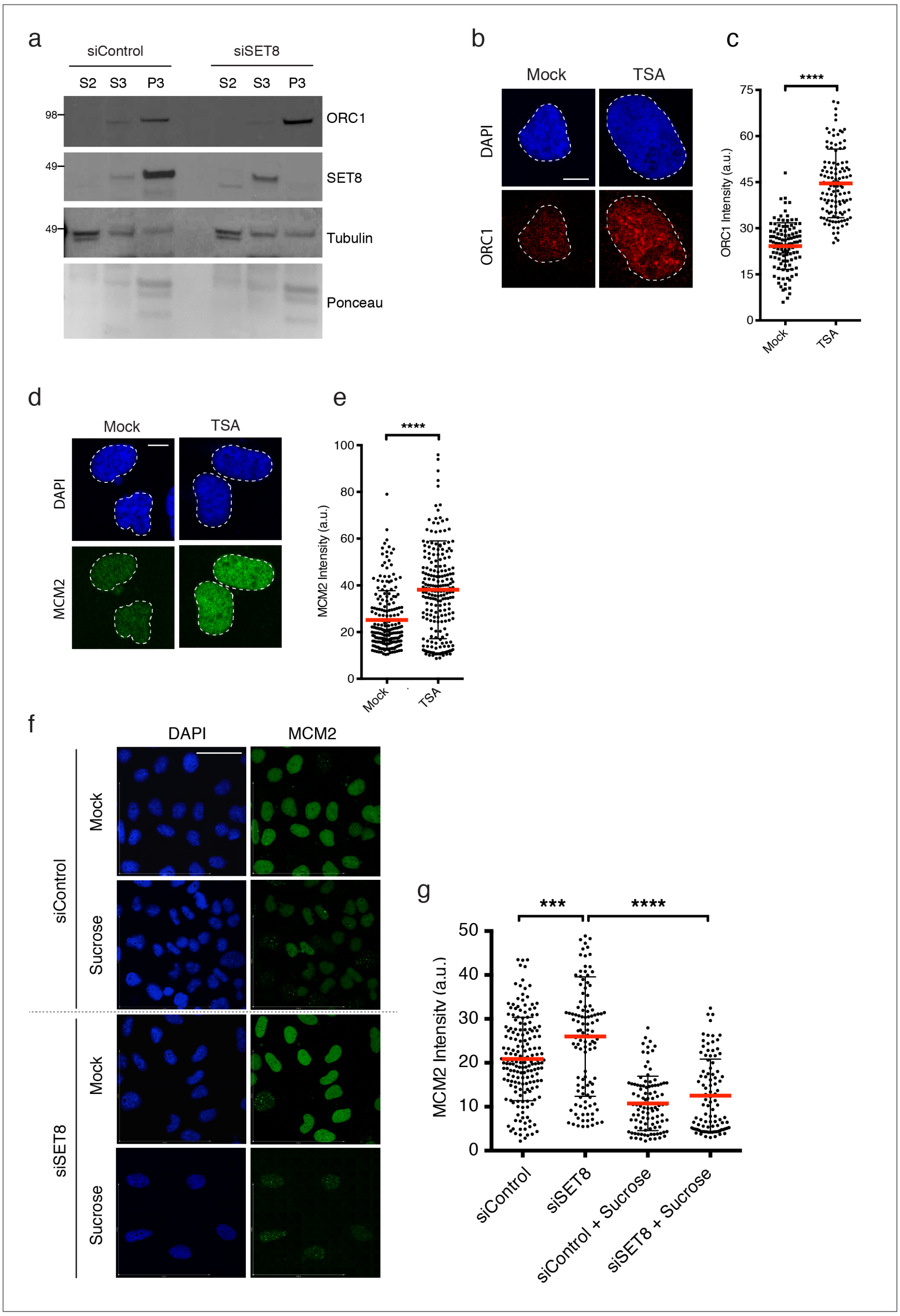

Supplementary Figure 7. TSA treated cells showed increased chromatin loading of licensing proteins while addition of sucrose reduced loading of MCM2-7 complex. **(a)** U2OS cells were synchronized with a double thymidine block and treated with either control or SET8 siRNA. Cells were released from the G1/S block, harvested and fractionated to obtain different cellular fractions as described in Materials and Methods. Samples were immunoblotted with the indicated antibodies. **(b)** U2OS cells were synchronized as in (Supplementary fig. 7a) and treated with HDAC inhibitor (TSA) at 12h. Cell were then fixed 3h later (15h post G1/S release) and stained with ORC1 antibody as well as DAPI for DNA after pre-extraction in CSK buffer containing 0,5% triton. (Bar, 10 μm). **(c)** Scatter plot showing the quantification of ORC1

intensity where mean ± SD indicated by solid lines. n>150, ****$p<0.0001$ (unpaired t-test). **(d)** U2OS cells were synchronized and treated as in (Supplementary fig. 7b). Cells were pre-extracted, fixed and immunostained with the MCM2 antibody as well as DAPI for DNA, after pre-extraction in CSK buffer containing 0,5% triton. (Bar, 10 µm). **(e)** Scatter plot showing the quantification of MCM2 intensity where mean ± SD indicated by solid lines. n>150, ****$p<0.0001$ (unpaired t-test). **(f)** U2OS cells synchronized by double thymidine block were transfected with Control and SET8 siRNA and were Mock or Sucrose treated at 12h post G1/S release for a total of 3h. Cells were then fixed at 15h post G1/S release and were immunostained with an MCM2 antibody after pre-extraction in CSK buffer containing 0,5% triton. (Bar, 50 µm). **(g)** Scatter plot showing the quantification of MCM2 intensity from cells in (A) where mean ± SD indicated by solid lines. n>100, ****$p<0.0001$ (unpaired t-test).

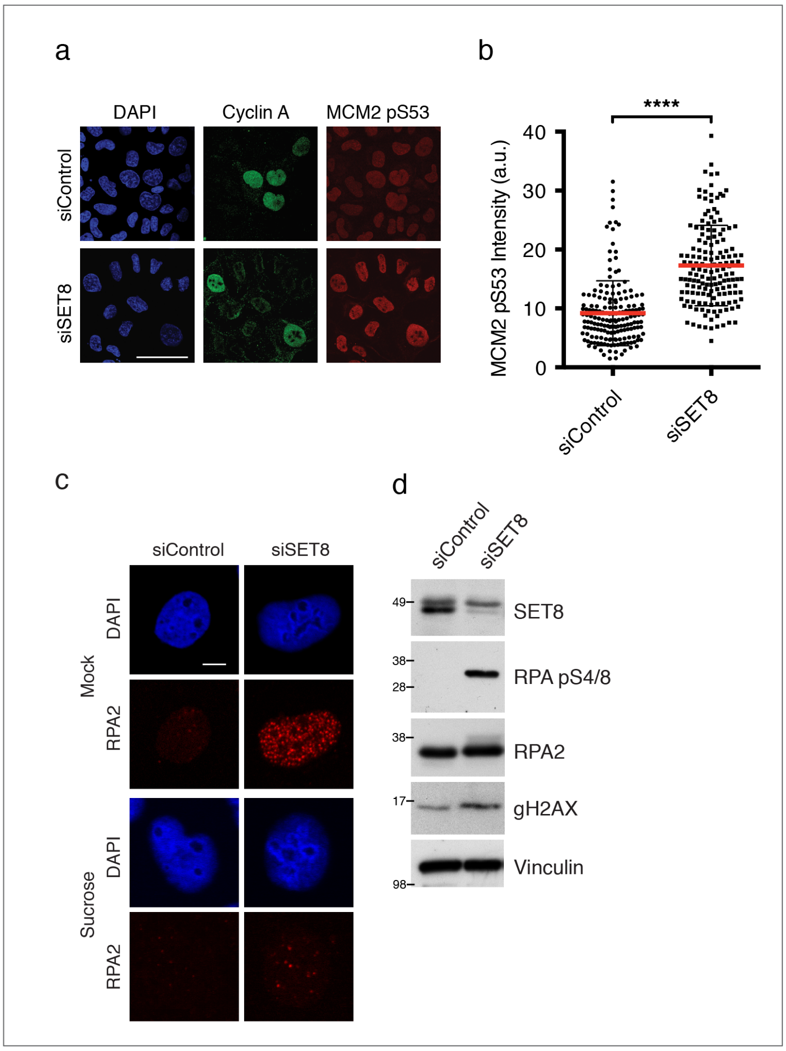

Supplementary Figure 8. siSET8 treated cells showed increased MCM2 phosphorylation and accumulation of ssDNA, which is reduced after treatment with sucrose. **(a)** U2OS cells were synchronized with double thymidine block and siRNA treated with siControl and siSET8. Cells were fixed at 15h post G1/S release and immunostained with the indicated antibodies. Bar, 50 μm. **(b)** Scatter plot showing the quantification of MCM2 phospho Serine 53 intensity from cells in (a) where mean ± SD indicated by solid lines. n>150, ****p<0.0001 (unpaired t-test). **(c)** U2OS cells synchronized with a double thymidine block were transfected with Control and SET8 siRNA and were Mock and Sucrose treated at 12h post G1/S release. Cells were then fixed at 15h post G1/S release and were immunostained with an RPA2 antibody after pre-extraction in CSK buffer containing 0,5% triton. Bar, 10 μm. **(d)** Chromatin was prepared from cells synchronized with a double thymidine block and siRNA treated with siControl and siSET8. Cells were harvested at 15h post G1/S release and samples were immunoblotted with the indicated antibodies.

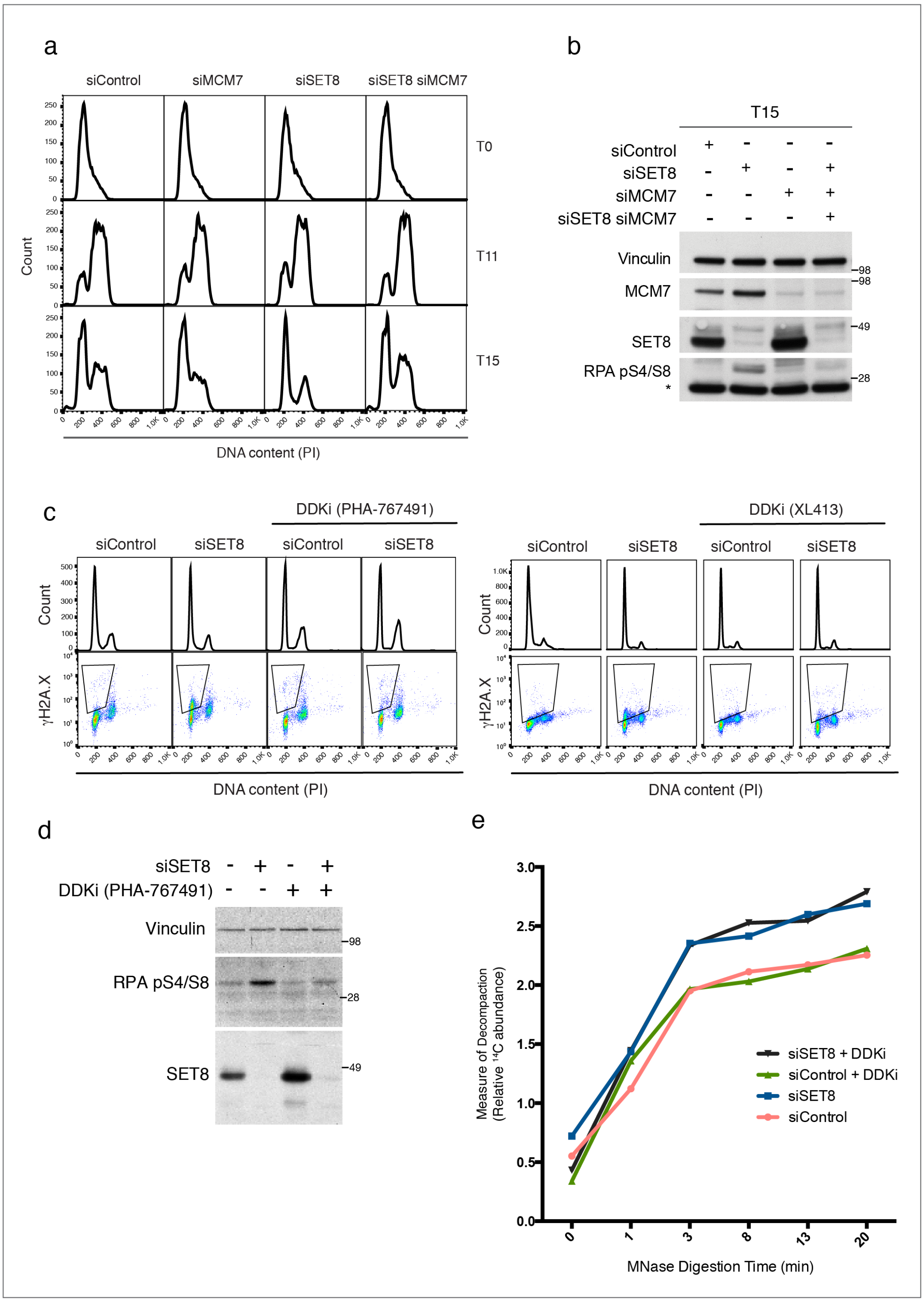

Supplementary Figure 9. MCM2-7 complex loading and activity are implicated in loss of genome stability in siSET8 treated cells. **(a)** U2OS cells were transfected with siMCM7 and synchronized with a double thymidine block. SET8 was depleted at the G1/S transition. Cells were then released and fixed at the indicated times, which correspond to G1/S, G2/M and G1 phases of the cell cycle. Graph shows PI (DNA) profile of the indicated samples. **(b)** Chromatin prepared from samples in (Supplementary fig. 9a) was immunoblotted with the indicated antibodies. * represents non-specific protein. **(c)** Flow cytometry profile of samples stained with the γH2A.X antibody and PI for DNA content from (Fig. 4f). Left: PHA-767491, right: XL413. **(d)** U2OS cells were synchronized with a double thymidine block and siRNA treated with siControl and siSET8. Cells were treated with DDKi (PHA-767491) at 11 h and were subsequently harvested at 15h

post G1/S release. Chromatin was prepared and immunoblotted with the indicated antibodies. **(e)** U2OS cells were synchronized with a double thymidine block and transfected with siControl and siSET8 at G1/S border. Thymidine containing ^{14}C was added throughout the cell culture. Cells were treated with DDKi (PHA-767491) at 11 h post G1/S release and harvested at 15 h post G1/S boundary. Nuclei were prepared and digested using MNase for varying duration of times. The graph represents the relative abundance of methyl-^{14}C released from chromatin after MNase digestion in control versus cells lacking SET8.

Fig. 1c

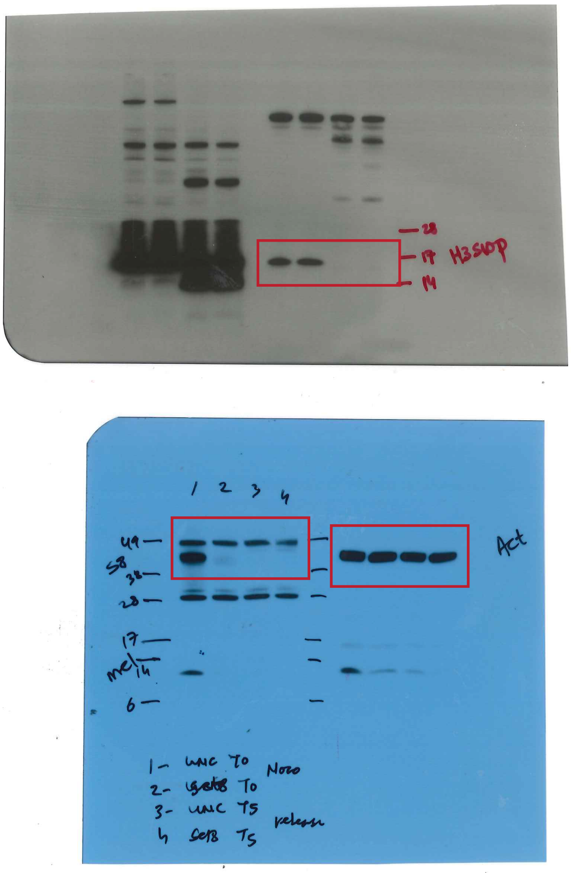

Fig. 3d

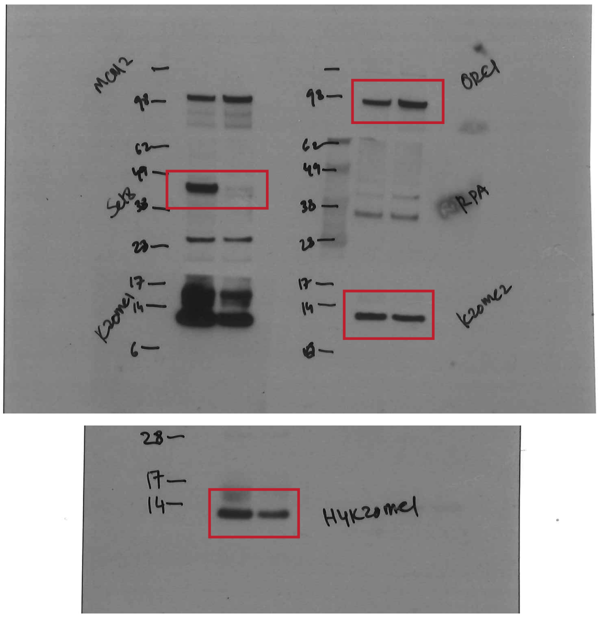

Fig. 2d

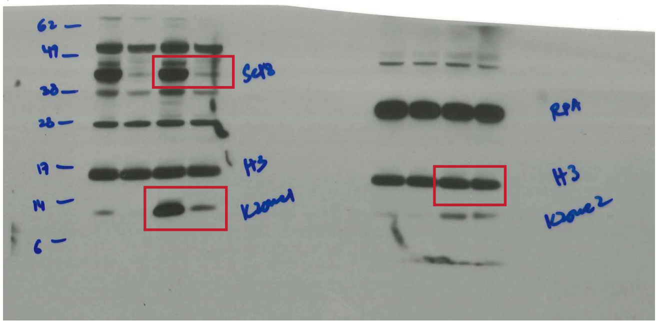

Supplementary Figure 10: Uncropped scans of immunoblots

Fig. 3e

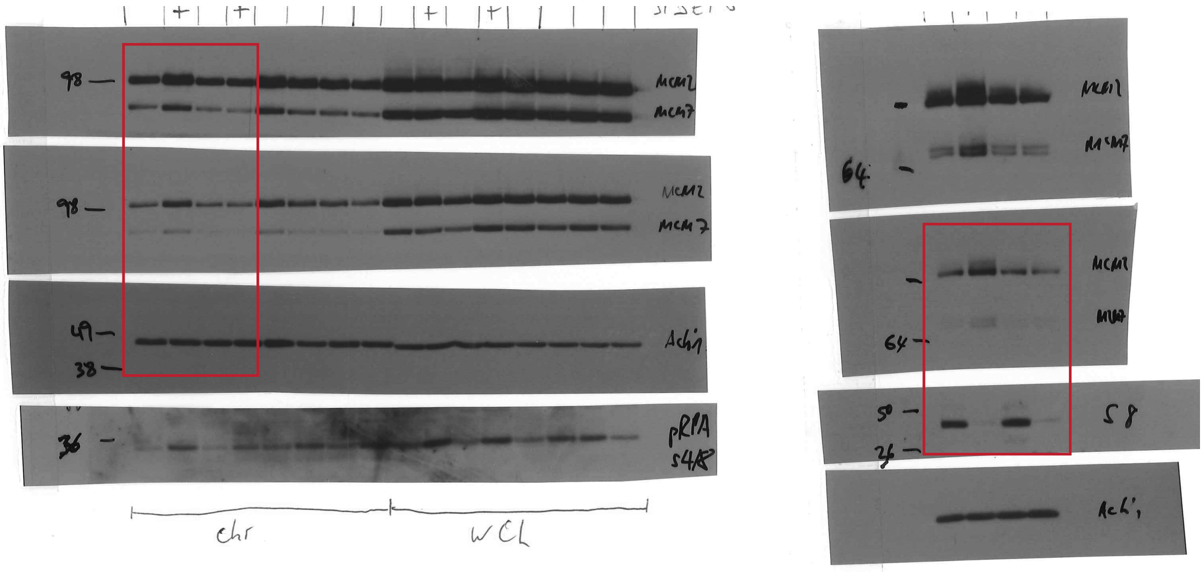

Supplementary Fig. 5c

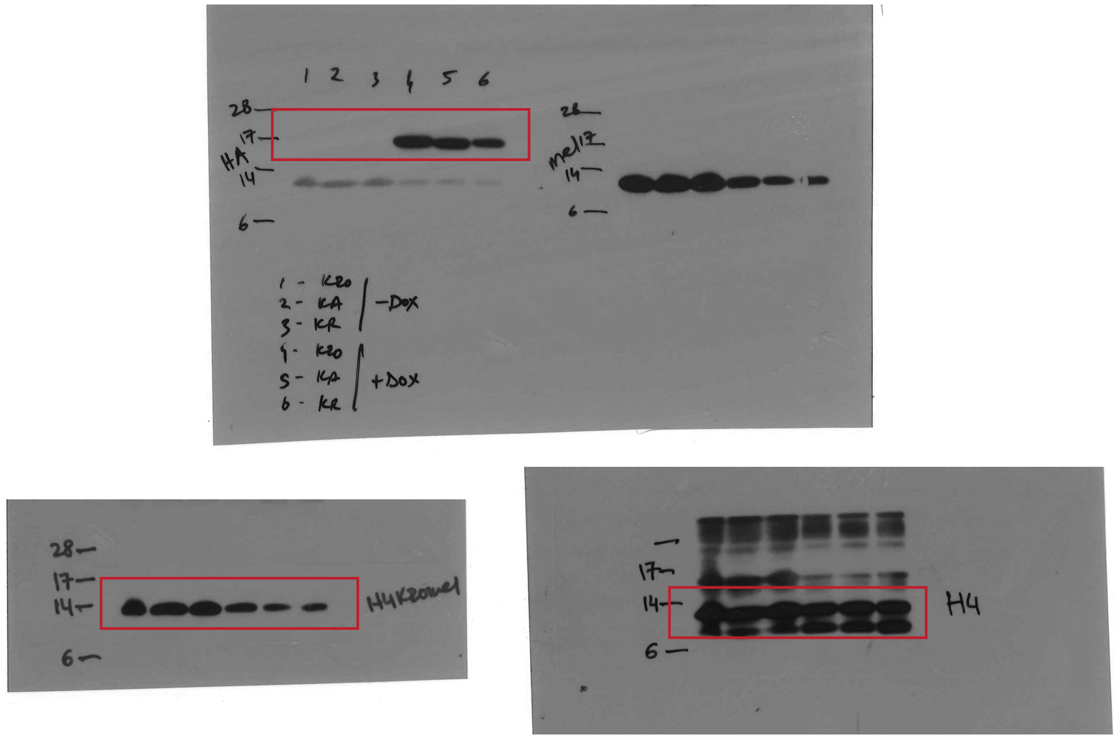

Supplementary Figure 10: Uncropped scans of immunoblots

Supplementary Fig. 7a

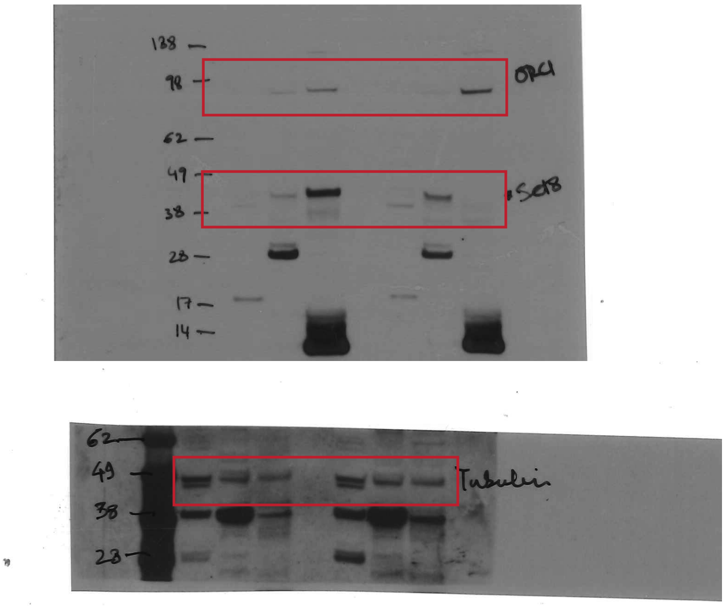

Supplementary Fig. 9b

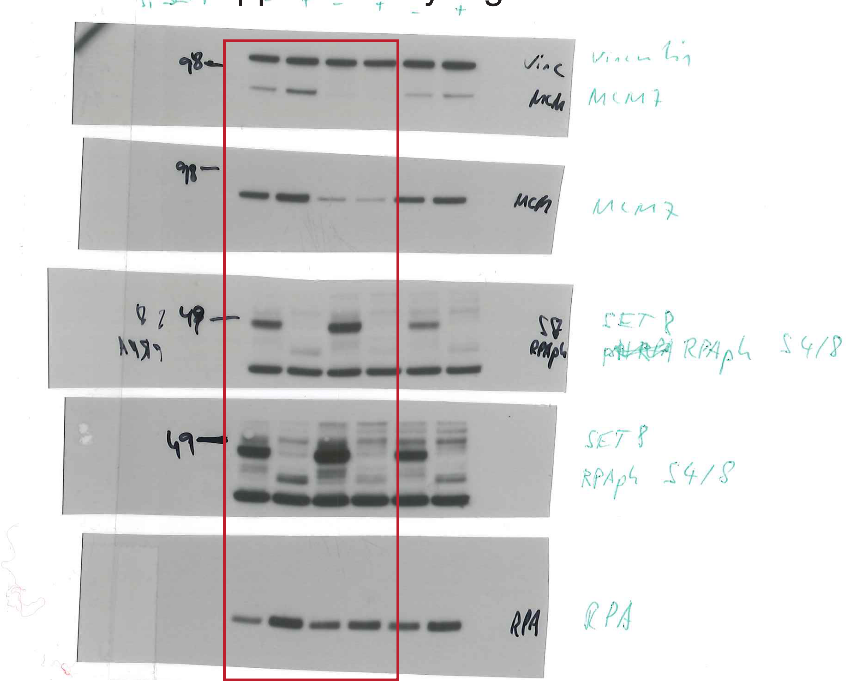

Supplementary Figure 10: Uncropped scans of immunoblots

Supplementary Table 1

List of primers

Name of primer	Sequence
plvx.F	CCCTCGTAAAGAATTCACTCTAGCGTTTAAACTTAAGCTT
plvx.R	GAGGTGGTCTGGATCCGCAGAATTCCACCACACTGGACTA
H4-F	ATGTCTGGGCGAGGTAAAGGTGGCAAGGGGCTG
H4-R	CAACCGCCGAAACCATAAAGGGTGCGACCCTGG
H4K20A-F	GCCAAGCGCCACCGGGCCGTGCTGCGGGACAAT
H4K20A-R	ATTGTCCCGCAGCACGGCCCGGTGGCGCTTGGC
H4K20R-F	GCCAAGCGCCACCGGCGCGTGCTGCGGGACAAT
H4K20R-R	ATTGTCCCGCAGCACGCGCCGGTGGCGCTTGGC
CMV-H4-F	ATGCACCGGTACCATGTCTGGCCGCGGCAAAGG
CMV-H4-R	ATGCGGAATTCATCCTCACTACTTGTCATCGTC

Supplementary Table 2

Flow Cytometry

Antibody	Species	Vendor	Catalogue number	Dilution
Anti-H3S10 phospho	Rabbit	Millipore	06-570	1/250
Anti-γH2A.X	Mouse	Millipore	05-636/JBW301	1/1000

Supplementary Table 3

Western Blotting

**Antibody**	**Species**	**Vendor**	**Catalogue number**	**Dilution**
Anti-SET8	Rabbit	Millipore	06-1304	1/1000
Anti-H3S10 phospho	Rabbit	Millipore	06-570	1/2000
Anti-Actin	Mouse	Millipore	MAB1501/C4	1/50000
Anti-H4K20me1	Mouse	Active Motif	39727/5E10-D8	1/1000
Anti-H4K20me1	Rabbit	Cell signaling	9724S	1/1000
Anti-Histone H3	Rabbit	Abcam	ab1791	1/25000
Anti-ORC1	Rabbit	Abcam	ab85830	1/1000
Anti-H4K20me2	Rabbit	Cell Signaling	9759S	1/1000
Anti-MCM2	Mouse	BD Transduction Lab	610700	1/2000
Anti-MCM7	Mouse	Santa Cruz	sc-9966	1/1000
Anti-Tubulin	Rabbit	Santa Cruz	sc-10732/H-183	1/2000
Anti-FLAG	Mouse	Sigma	F3165	1/1000
Anti-HA	Mouse	Covance	MMS101P	1/1000
Anti-H4	Rabbit	Millipore	05-858/62-141-13	1/5000
Anti-γH2A.X	Mouse	Millipore	05-636/JBW301	1/1000
Anti-RPAS4/8 phospho	Rabbit	Bethyl Labs	A300-245A	1/1000

Supplementary Table 4

Immunofluorescence Microscopy

Antibody	Species	Vendor	Catalogue number	Dilution
Anti-γH2A.X	Mouse	Millipore	05-636/JBW301	1/700
Anti-ORC1	Rabbit	Abcam	ab85830	1/500
Anti-MCM2	Mouse	BD Transduction Lab	610700	1/300
Anti-BrdU	Mouse	Amersham	RPN20AB	1/200
Anti-RPA	Mouse	Millipore	NA19L-100U/Ab3	1/300
Anti-HA	Mouse	Covance	MMS101P	1/1000
Anti-MCM2S53 phospho	Rabbit	Bethyl Labs	A300-756A	1/1000

Supplementary Table 5

Secondary Antibodies

Antibody	Vendor	Catalogue number	Dilution
HRP Goat Anti-Rabbit IgG Antibody (Peroxidase)	Vector Laboratories	PI-1000	1/5000
HRP Horse Anti-Mouse IgG Antibody (Peroxidase)	Vector Laboratories	PI-2000	1/5000
Donkey anti-Rabbit IgG (H+L) Highly Cross-Adsorbed Secondary Antibody, Alexa Fluor 488	ThermoFisher Scientific	A-21206	1/1000
Goat anti-Mouse IgG (H+L) Cross-Adsorbed Secondary Antibody, Alexa Fluor 488	ThermoFisher Scientific	A-11001	1/1000
Donkey anti-Rabbit IgG (H+L) Highly Cross-Adsorbed Secondary Antibody, Alexa Fluor 594	ThermoFisher Scientific	A-21207	1/1000
Donkey anti-Mouse IgG (H+L) Highly Cross-Adsorbed Secondary Antibody, Alexa Fluor 594	ThermoFisher Scientific	A-21203	1/1000
